# Circular RNA as a Potential Biomarker for Melanoma: A Systematic Review

**DOI:** 10.3389/fcell.2021.638548

**Published:** 2021-04-01

**Authors:** Keyun Tang, Hanlin Zhang, Yaqi Li, Qiuning Sun, Hongzhong Jin

**Affiliations:** State Key Laboratory of Complex Severe and Rare Diseases, Department of Dermatology, Peking Union Medical College Hospital, Chinese Academy of Medical Science and Peking Union Medical College, Beijing, China

**Keywords:** circular RNA, melanoma, biomarker, therapeutic targets, systematic review

## Abstract

Circular RNAs (circRNAs) are newly discovered RNAs with covalently looped structures. Due to their resistance to RNAase degradation and tissue-specific expression, circRNAs are expected to be potential biomarkers in early diagnosis and target treatment of many diseases. However, the role of circRNAs in melanoma still needs to be systematically reviewed for better understanding and further research. Based on published articles in PubMed, Embase, Cochrane Library, and Web of Science database, we systematically reviewed the implications and recent advances of circRNAs in melanoma, focusing on function, mechanism, and correlation with melanoma progression. According to inclusion and exclusion criteria, a total of 19 articles were finally included in this systematic review. Of the 19 studies, 17 used human samples, including melanoma tissues (*n* = 16) and blood serum of patients with melanoma (*n* = 1). The sample size of the study group ranged from 20 to 105 based on the reported data. Several studies explored the association between circRNAs and clinicopathological characteristics. circRNA dysregulation was commonly observed in melanoma patients. circRNAs function in melanoma by miRNA sponging and interaction with RNA binding proteins (RBP), ultimately controlling several important signaling pathways and cancer-related cellular processes, including proliferation, migration, invasion, metastasis, apoptosis, and glucose metabolism. circRNA expression could be associated with prognostic factors and drug responses, consolidating the potential clinical value in melanoma. Herein, we clarified the functional, prognostic, and predictive roles of circRNAs in melanoma in this systematic review, providing future directions for studies on melanoma-associated circRNAs.

## Introduction

Melanoma accounts for a small group of skin cancer diagnosed each year (3%) in contrast to a huge number of skin cancer deaths (65%) (Pavri et al., 2016). Melanoma originates in the melanocytes in the basal cell layer of the epidermis as a consequence of genetic mutation mainly caused by ultraviolet exposure. Skin pigmentation phenotype, family and personal history of malignancies, and the existence of atypical nevi are considered predisposing factors (Mohammadpour et al., 2019; Zhang et al., 2021). Early-stage melanoma can be effectively controlled by surgical resection. However, melanoma is associated with high metastasis potential and resistance to conventional therapies such as radiotherapy and chemotherapy, thereby leading to a poor prognosis (Payandeh et al., 2019). As reported, the 5-year overall survival rate of patients with aggressive invasion is no more than 5% (Cummins et al., 2006). Despite the rapid development of diagnostic and therapeutic techniques, the treatment and prognosis of patients with melanoma remain unsatisfied. An incomplete understanding of genetic variation and molecular mechanisms of melanoma occurrence and progression partly leads to a poor prognosis. Thus, it is essential to unravel the pathogenesis of melanoma to find sensitive biomarkers and targeted therapy sites.

Covalently closed circular RNAs (circRNAs) are a large class of endogenous RNA splicing products that emerge as essential regulators in recent years. circRNAs appear to be conserved across multiple species and exhibit development stage-dependent and tissue-specific expression patterns. It forms a continuous loop structure joining by the 3′ and 5′ ends with neither a free 5′ end cap nor a 3′ polyadenylated tail. circRNAs show high resistance to RNAase degradation compared to linear RNAs, displaying high stability and abundance in the cell.

**Figure 1:**
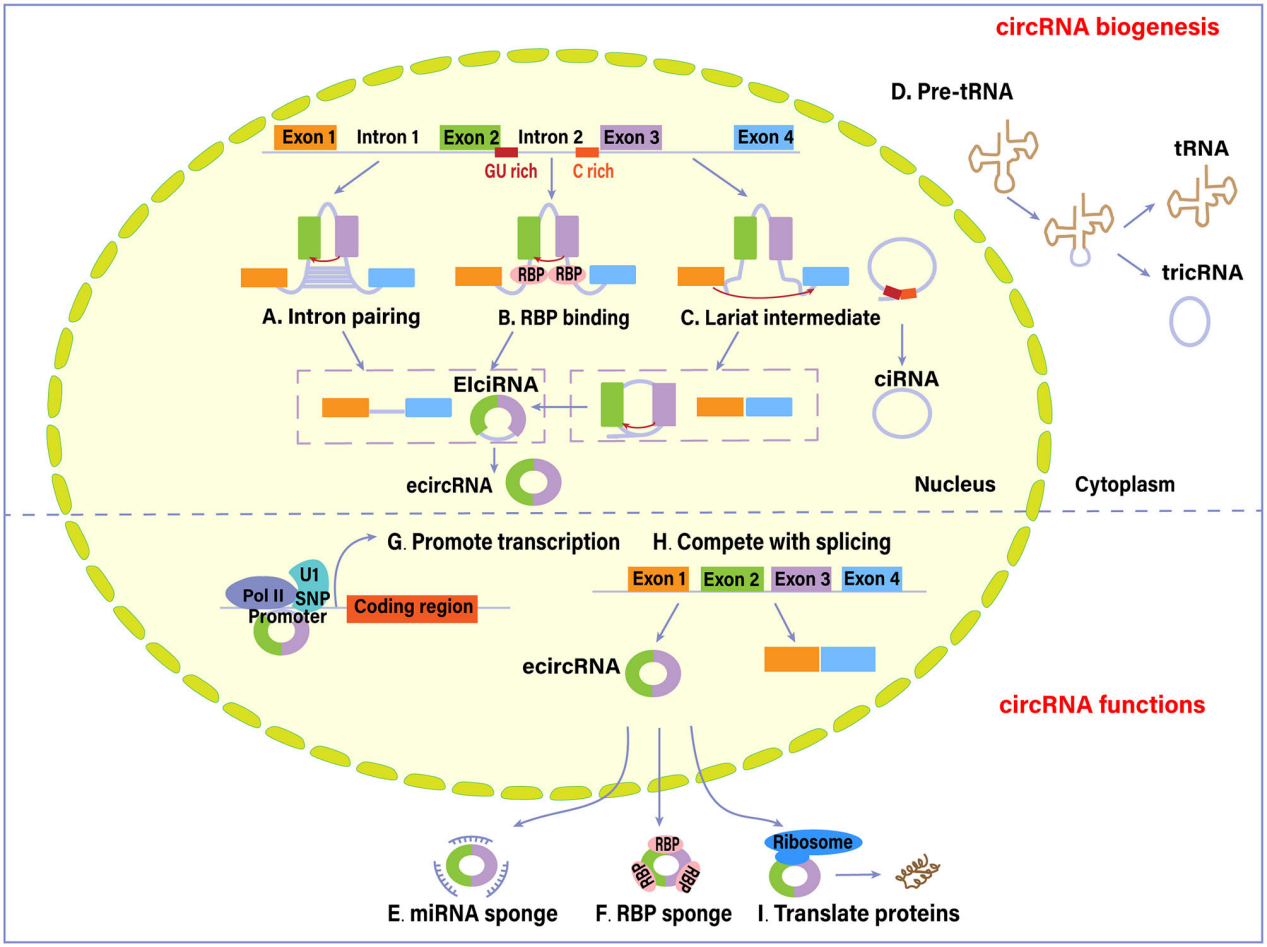
Biogenesis and functions of circular RNAs (circRNAs). Four different mechanisms mainly account for the biogenesis of circRNAs: **(A)** intron pairing pathway, **(B)** RNA-binding proteins (RBPs)-associated pathway, **(C)** lariat-driven pathway, and **(D)** synthesized from introns spliced from pre-tRNA. As of functions, **(E)** circRNAs can sequester and absorb miRNAs to regulate the function of miRNAs. **(F)** circRNAs interact with RBPs and thus regulate their cellular localization and activity. **(G)** circRNAs interact with RNA polymerase II (Pol II) or U1 small nuclear ribonucleoprotein (snRNP) in the promoter region to promote the transcription of their host genes. **(H)** circRNAs compete with canonical splicing of pre-mRNAs to regulate gene expression. **(I)** circRNAs are also capable of encoding proteins. EIci-RNA, exon-intron circRNA; ecircRNA, exonic circRNA (Hansen et al., 2013; Chen and Yang, 2015; Li et al., 2015; Wan et al., 2016; Liu et al., 2017; Meng et al., 2017; Noto et al., 2017; Han et al., 2018; Shen et al., 2019; Verduci et al., 2019; Wawrzyniak et al., 2020).

circRNA biogenesis is dependent on canonical splicing signal and canonical splicing machinery. cicRNAs are mainly categorized into four types based on their origin: exonic circRNAs (ecircRNAs), exon-intron circRNAs (EIciRNAs), circular intronic RNAs (ciRNAs), and tRNA intronic circular RNAs (tricRNAs), which are produced through different mechanisms (Meng et al., 2017). Two possible models for ecircRNAs and EIciRNAs formation are “direct back-splicing” model and “exon skipping” or “lariat intermediate” model (Verduci et al., 2019). In the former model, a looping structure is formed via base-pairing between inverted repeat elements (e.g., Alu repeats) across exon-flanking introns (Figure 1A), or via the dimerization of RNA- binding proteins (RBPs) that bind to specific motifs in the flanking introns, such as protein quaking (QKI) and Muscleblind (MBL) (Figure 1B). This processing is followed by back-splicing of pre-mRNAs for a circRNA together with an exon-intron(s)-exon intermediate. In the latter one, several exons are kept in a linear mRNA product during canonical splicing, while alternative exons and lariat introns are spliced out and back-spliced further to form a circRNA (Chen and Yang, 2015). The introns within circular RNAs can be completely removed through internal splicing or not, ultimately producing ecircRNAs or EIciRNAs. Intron lariat that escapes from normal debranching and degradation processing leads to the formation of ciRNAs. Its biogenesis depends on consensus motifs located on both sides of the introns (a GU-rich motif near the 5′-splicing site and a C-rich motif near the branchpoint site) (Figure 1C). There is another conserved mode of RNA circularization occurring in eukaryotes and archaea, in which the splicing endonuclease complex recognizes the sequence motif of pre-tRNAs and splice the tRNA introns (Noto et al., 2017). The intron termini are subsequently joined to form a tRNA intronic circular RNA (tricRNA), while the exon halves are ligated to form a mature tRNA (Figure 1D).

circRNAs can alter gene expression via different regulatory modes. Several circRNAs containing miRNA response elements act as a miRNA sponge (Figure 1E) (Han et al., 2018). The binding of circRNAs to target miRNAs causes inhibition of miRNA function. ciRS-7 circRNA (also known as CDR1as), one of the most studied circRNAs, has high sponging activity for miRNA, particularly for miR-7 (Hansen et al., 2013). An increasing number of studies in the context of cancer have attributed pro-oncogenic effects to this circRNA. Circ-ITCH could enhance the expression of its parental cancer suppressor gene ITCH by sponging oncogenic miR-7 and miR-214 and suppress the Wnt/β-catenin signaling (Wan et al., 2016).

circRNAs have sponging effects for RNA binding proteins (RBP) (Figure 1F). circRNAs could bind, sequester, or transport RBPs to particular subcellular compartments (Wawrzyniak et al., 2020). For example, circ-Amotl1 could bind to c-myc, PDK1, STAT3, and AKT1, facilitating their translocation into nucleus and regulating the expression of target genes (Han et al., 2018; Wang M. et al., 2018) circRNAs are capable of forming protein scaffolds for the assembly of other compounds and protein complexes. Besides, circRNAs and their cognate mRNA competitively bind to RBP, which modulates the expression of target genes. The combination of circPABPN1 and HuR (a transcriptional activator of RBP) prevents the HuR binding to PABPN1 mRNA, subsequently suppressing PABPN1 translation (Abdelmohsen et al., 2017).

Other identified gene regulation modes are based on circRNAs capacity to interact with the transcription machinery. EIciRNAs are revealed to interact with RNA Polymerase II and U1 small nuclear ribonucleoprotein in the promoter region of the target gene (Figure 1G) (Li et al., 2015). In addition, the circularization of circRNA molecules could negatively affect canonical splicing of pre-mRNAs into corresponding linear RNAs, since they share the same splice sites and spliceosomal machinery (Figure 1H). Therefore, circRNA formation results in a decreased production of canonical proteins by competing with linear splicing (Liu et al., 2017).

Being translated into functional proteins is another function of circRNAs, although most of them are deemed as non-coding RNAs (Figure 1I) (Shen et al., 2019). circRNAs may contain a translation start codon and ribosome entry sites. circ-ZNF609, which functions in myoblast proliferation, can be translated into proteins *in vivo* through a cap-independent and splicing-dependent mechanism (Legnini et al., 2017). Pamudurti et al. (2017) provided evidence of a set of EIcircRNAs that interacted with ribosomes and encoded proteins in a cap-independent way.

Emerging evidence shows that circRNAs may exert regulatory functions in multiple human diseases as they exhibit aberrant expression in a variety of cancers, such as gastric cancer (Wang and Dong, 2019), lung cancer (Drula et al., 2020), and hepatocellular carcinoma (Wang M. et al., 2018). These circular transcripts may play a role in several cancer-related physiological changes such as apoptosis, vascularization, invasion, and metastasis.

This systematic review outlines the recent findings on deregulation and the possible function of circRNAs in melanoma. We also discussed its potential as novel biomarkers, therapeutic targets, and in other clinical implications.

## Methods

This systematic review was designed in line with the standard criteria Preferred Reporting Items for Systematic Reviews and Meta-Analysis (PRISMA) (Moher et al., 2009). A systematic review search was conducted on PubMed, Embase, Cochrane Library, and Web of Science database to identify eligible publications on February 21st, 2021. The keywords included melanoma, circularRNA, circRNA, and Circular RNA. Two researchers (Tang, K and Zhang, H) conducted an exhaustive search independently to select articles and reached a consensus based on the suggestions of a senior expert (Jin, H). Relevant articles were identified during the screening process based on titles and abstracts. Then duplicates were removed, and full texts of these articles were analyzed, from which we selected the ones eligible for inclusion in the systematic review.

We included studies meeting the following criteria: studies investigating the biological functions or/and potential pathways of circRNAs in melanoma; studies investigating the expression of circRNAs between melanoma tissues or cancer cells and normal tissues; studies discussing the potential role of circRNAs as a biomarker for early diagnosis; studies investigating circRNAs with clinical implications of melanoma. Only articles written in English were included. Review, comments, articles in other languages, studies exploring circRNA function in other cancer types or diseases, studies using bioinformatics methods alone, studies only focusing on circRNA biogenesis or function were all excluded. From the eligible articles with original data, we extracted the necessary information, including the location of study, name of the first author, publication year, type of melanoma, type of detected samples/cell line, number of melanoma tissues and normal controls, dysregulated circRNAs in melanoma, downstream proteins and signaling pathways, and clinical implications. One author (Tang, K) used the Newcastle–Ottawa Scale (NOS) to assess the methodological quality and risk of bias of the included studies if applicable (Wells et al., 2014; Mu et al., 2021). The NOS evaluated seven items in three categories, including Selection, Comparability of cases and controls, and Exposure. A maximum of nine stars can be awarded to a study, and a study with no less than five stars can be considered a high-quality one (Mu et al., 2021).

## Results

### Search Results

**Figure 2:**
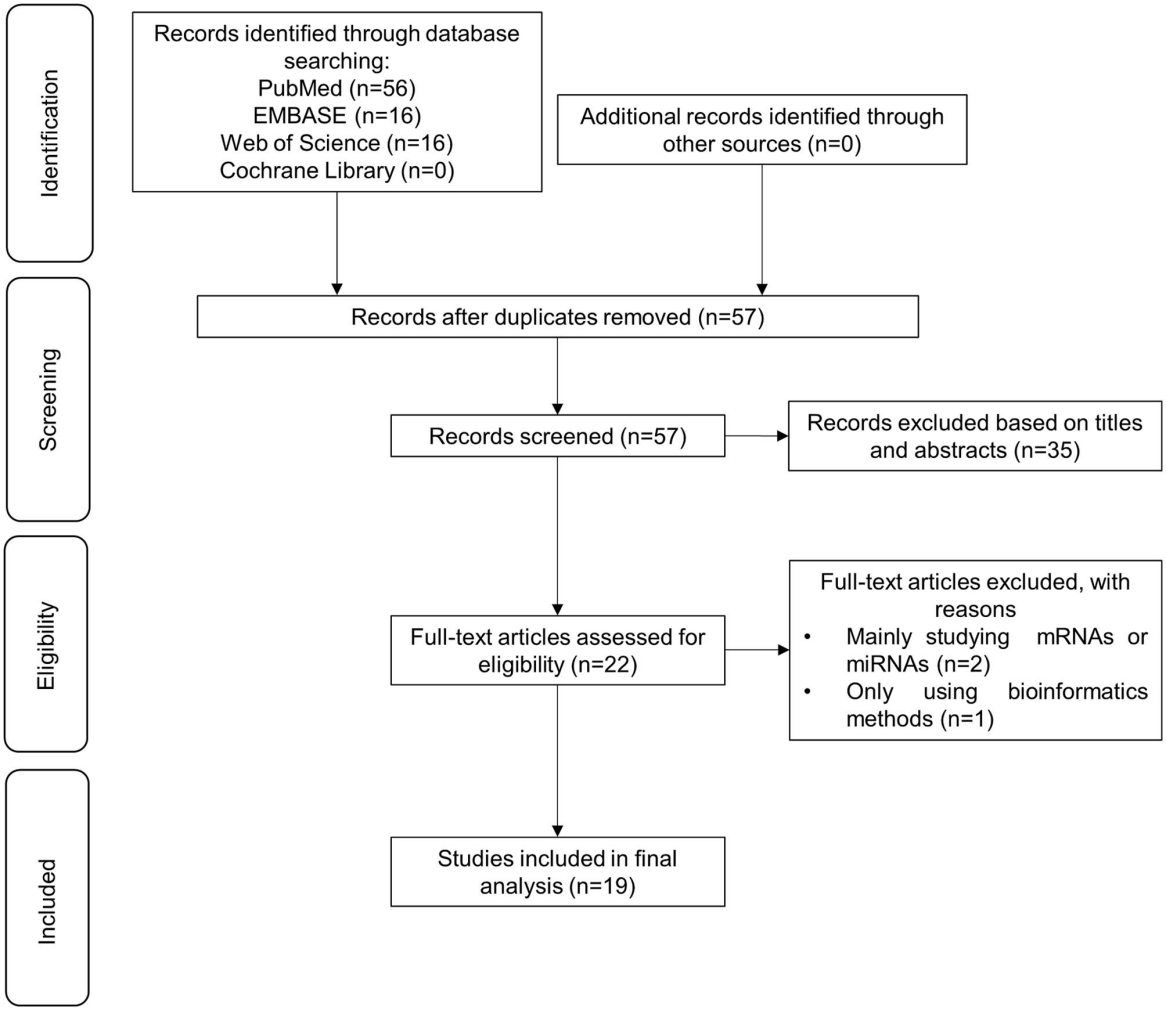
Flow chart depicting the selection process of 16 studies included in this systematic review.

A total of 88 articles were identified with the searching strategy. Duplicates and irrelevant articles were removed during the selection process. Two researchers independently screened articles based on inclusion and exclusion criteria. The full-text review yielded 22 articles. We excluded two studies because their main focus was not circRNAs, and one because it used bioinformatics methods alone. A total of 19 articles were finally included in this systematic review. Details of the selection process are presented in Figure 2. The 19 articles discussed the expression pattern, molecular mechanisms, and clinical implication in different types of melanomas, including cutaneous melanoma (*n* = 4), uveal melanoma (*n* = 2), conjunctiva melanoma (*n* = 1), and oral mucosal melanoma (*n* = 1). The other 11 did not specify a melanoma type. The majority of studies were conducted in China (*n* = 18). Of the 19 studies, 17 used human samples, including melanoma tissues (*n* = 16) and blood serum of patients with melanoma (*n* = 1). The sample size of the study group ranged from 20 to 105 based on the reported data. The methodological quality of these 17 studies involving human subjects was evaluated as high-quality. The remaining two studies only used normal cell lines and melanoma cell lines with different metastasis, and the methodological quality assessment was not applicable. Details for the methodological quality and risk of bias were provided in Supplementary Table 1. circRNAs types, expression patterns, associated molecules, and biological action of these studies were summarized in Table 1.

**Table 1:** Melanoma-associated circRNAs in the included articles.

**Melanoma type**	**Circular RNA**	**Expression level**	**Target miRNA or proteins**	**Downstream proteins and signaling pathways**	**Function/clinical association**	**Model**	**Sample/cell line**	**Methodological quality**	**Location**	**References**
Uveal melanoma	circ_0119873 circ_0128533 circ_0047924 circ_0103232 circRNA10628-6	Up	ND	ND	ND	Human	20 melanomas vs. 20 normal tissues	Good	China	Yang et al., 2018
	circ_0032148 circ_0133460	Down	ND	ND	ND					
Metastatic melanoma	circ_0000082 circ_0016418	Up	ND	ND	Promotes cell proliferation, migration and invasio	*In vitro*	WM35 and WM451	NA	China	Wang M. et al., 2018
	circ_0023988 circ_0008157 circ_0030388	Down	ND	ND	Inhibits cell proliferation, migration and invasion					
Metastatic oral mucosal melanoma	circ_0005320 circ_0067531 circ_0008042	Up	ND	GTPase^†^ MAPK pathway^†^ Translation repressor^†^	Correlates with metastasis^†^	Human	30 melanomas vs. 30 normal tissues	Good	China	Ju et al., 2018
	circ_0000869 circ_0000853	Down	ND	G-protein coupled receptor pathway^†^ NF-kappa B pathway^†^	Correlates with metastasis^†^					
Melanoma	circ_0084043	Up	miR-153-3p	Snail	Promotes cell proliferation, migration and invasion; correlates with TNM stage and overall survival	*In vitro, in vivo*, human	A375, SK-MEL-1, SK-MEL-5 and A875; mice; 33 melanomas vs. 33 normal tissues	Good	China	Luan et al., 2018
Melanoma	circ_0025039	Up	miR-198	CDK4	Promotes cell proliferation, invasion and glycolysis; correlates with TNM stage and overall survival	*In vitro*, human	A375, SK-MEL-1, A2058 and 293T; 43 melanomas vs. 18 paired normal tissues	Good	China	Bian et al., 2018
Conjunctival melanoma	circ_0083444 (circ-MTUS1)	Up	miR-622, miR-1208^†^	ErbB pathway, MAPK pathway, and Wnt pathway^†^	Promotes cell proliferation	*In vitro, in vivo*, human	CRMM-1, CRMM-2 and CM2005.1; NOD/SCID mice; not mentioned	Good	China	Shang et al., 2019
Melanoma	circ-ITCH	Down	ND	GLUT1	Promotes cell proliferation and glucose uptake	*In vitro*, human	A375 and M21; 56 melanomas vs. 56 normal tissues	Good	China	Lin et al., 2019
Cutaneous melanoma	circ_0016418	Up	miR-625	YY1	Promotes cell proliferation, migration, invasion and epithelial to mesenchymal transition	*In vitro*, human	SKMEL1 and SKMEL5; 30 melanomas vs. 30 normal tissues	Good	China	Zou et al., 2019
Melanoma	circ_0020710 (circ-CD151)	Up	miR-370-3p	CXCL12	Promotes cell proliferation, migration and invasion; correlated with advanced Breslow depth, Clark level, cytotoxic lymphocyte exhaustion and anti-PD-1 therapy resistance	*In vitro, in vivo*, human	A375, A2058, A875, Sk-mel-28, MV3, M14; C57BL/6 mice; 88 melanomas vs. 88 normal tissues and 18 benign nevi tissues	Good	China	Wei et al., 2020
Melanoma	circ_0002770	Up	miR-331-3p	DUSP5/TGFBR1 axis	Promotes cell proliferation, invasion and migration; correlated with a poor prognosis	*In vitro, in vivo*, human	SKMel1,A375 and A875; nude mice; 20 melanomas vs. 20 normal tissues	Good	China	Qian et al., 2020
Melanoma	circ-FOXM1	Up	miR-143-3p	FLOT2	Promotes cell proliferation, invasion and glycolysis; inhibits apoptosis	*In vitro, in vivo*, human	A2058 and A375; BALB/c nude mice; 30 melanomas vs. 30 normal tissues	Good	China	Tian et al., 2020
Primary cutaneous melanoma	circ_0084043	Up	miR-429	TRIB2; Wnt/β- catenin pathway	Promotes cell proliferation, migration and invasion; inhibits apoptosis	*In vitro, in vivo*, human	A375 and SK-MEL-28, BALB/C-nude mice; 30 melanomas vs. 30 normal tissues	Good	China	Chen J. et al., 2020
Melanoma	circ_0085533 (circ-MYC)	Up	miR-1236	LDHA	Promotes cell proliferation, glycolysis, and lactate production	*In vitro*, human	Mel-CV and Mel-RM; 25 melanomas vs. 25 normal tissues	Good	China	Jin et al., 2020
Cutaneous melanoma	ciRS-7 (CDR1as)	Down	IGF2BP3	SNAI2, MEF2C, etc.	Promotes cell invasion and metastasis; correlates with progression-free, overall survival and distinct therapeutic responses	*In vitro, in vivo*, human	501MEL and etc.; mice; 105 (53 primary melanomas vs. 52 metastatic melanomas)	Good	The United States	Hanniford et al., 2020
Melanoma	circ_0027247 (circ-GLI1)	Up	RPS6KB2 (p70S6K2)	Hedgehog/GLI1 pathway and Wnt/β-catenin pathway	Promotes cell migration, invasion, angiogenesis and metastasis	*In vitro, in vivo*	A375, MEL-RM, B16, M14, SK-MEL-2, SK-MEL-28; BALB/c nude mice	NA	China	Chen J. et al., 2020
Melanoma	circ_0016418	Up	miR-605-5p	GLS	Promotes cell proliferation, metastasis, glutamine catabolism and tumor growth; inhibits cell cycle arrest and apoptosis	*In vitro, in vivo*, human	HEMn-LP, A375 and A875; BALB/c nude mice; 30 melanoma vs. 30 normal tissues	Good	China	Lu et al., 2020
Cutaneous melanoma	circ_0079593	Up	miR-516b	GRM3	Promotes cell proliferation, metastasis, glucose metabolism; inhibits apoptosis		PIG1, A357 and SK-MEL-2; BALB / c-nude mice; 41 melanomas vs. 41 normal tissues	Good	China	Lu et al., 2020
Melanoma	circ_0001591	Up	miR-431-5p	ROCK1/PI3K/AKT	Promotes cell proliferation and invasion; inhibits apoptosis; correlates with overall survival and disease-free survival	*In vitro*, human	A2058; 53 blood serums of patients with melanoma vs. 53 blood serums of healthy controls	Good	China	Yin et al., 2021
Uveal melanoma	circ_0119872	Up	miR-622	G3BP1; Wnt/β-catenin pathway and mTOR pathway	Promotes cell proliferation, angiogenesis and tumor growth	*In vitro, in vivo*, human	APRE-19, SP6.5, VUP, OCM-1, 92–1, OCM-1A, MUM-2B, and OM431; BALB/c-nude mice; not mentioned	Good	China	Liu et al., 2021

† *Only predicted by bioinformatics analysis*. *ND, not determined; NA, not applicable*.

### Expression Patterns of circRNAs in Melanoma

**Figure 3:**
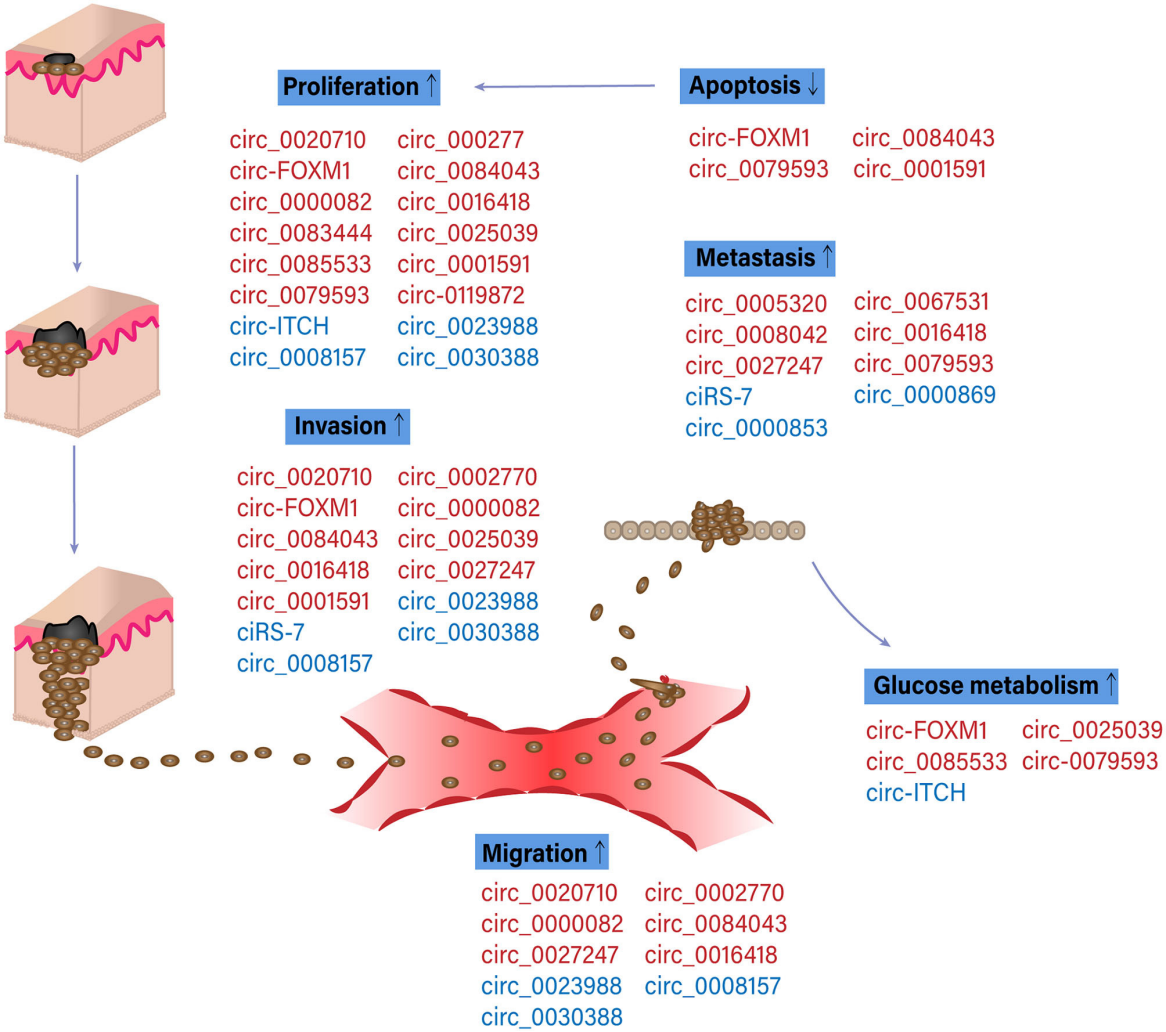
Functions of specific circular RNAs (circRNAs) in melanoma pathogenesis. Schematic diagram of circRNAs involved in melanoma progression. Red and blue color indicated the oncogenic and suppressive functions of circRNAs in different biological processes involved in melanoma progression, respectively. Up and down arrows indicated the activation and inhibition of physiological processes in melanoma pathogenesis, respectively (Bian et al., 2018; Ju et al., 2018; Luan et al., 2018; Wang Q. et al., 2018; Lin et al., 2019; Shang et al., 2019; Zou et al., 2019; Chen J. et al., 2020; Chen Z. et al., 2020; Hanniford et al., 2020; Jin et al., 2020; Lu and Li, 2020; Lu et al., 2020; Qian et al., 2020; Tian et al., 2020; Wei et al., 2020; Liu et al., 2021; Yin et al., 2021).

The potential functions of circRNAs in melanoma development and progression were presented in Figure 3. The first study concerning the expression pattern of circRNAs in melanoma was conducted by Wang Q. et al. (2018). Melanocytes were used in this study as a control. circRNA microarray revealed that five circRNAs were overexpressed, and four circRNAs were downregulated in both the low-metastatic melanoma cell line (WM35) and the high-metastatic cell line (WM451). In functional tests, circ_0023988, circ_0008157, or circ_0030388 knockdown considerably promoted the proliferation and invasion of WM35 cells while the silencing of circ_0000082 or circ_0016418 inhibited the propagation and invasion of WM451 cells.

Other studies investigating the expression pattern of circRNAs used normal tissues as control. Ju et al. (2018) analyzed circRNAs expression profile in 6 oral mucosal melanoma (OMM) samples with lymph node dissemination. Among 90 significantly dysregulated circRNAs, circ_0005320, circ_0067531, circ_0008042 were remarkably upregulated in contrast with normal tissues and non-metastatic lymph nodes, whereas circ_0000869 and circ_0000853 were relatively downregulated. Bioinformatics predictions, Gene Ontology (GO) and pathway analyses demonstrated that these differentially expressed circRNAs might participate in cellular metabolism, serve as miRNA-sponge, and regulate OMM tumorigenesis and metastatic through circRNA-miRNA axis. Similar experiments in uveal melanoma showed that a total of 50,579 circRNAs were differentially expressed between uveal melanoma tissues and normal ones, providing promising targets for future research on diagnosis and treatment (Yang et al., 2018).

### Roles in Molecular Mechanisms of Melanoma

#### Sponging miRNAs

**Figure 4:**
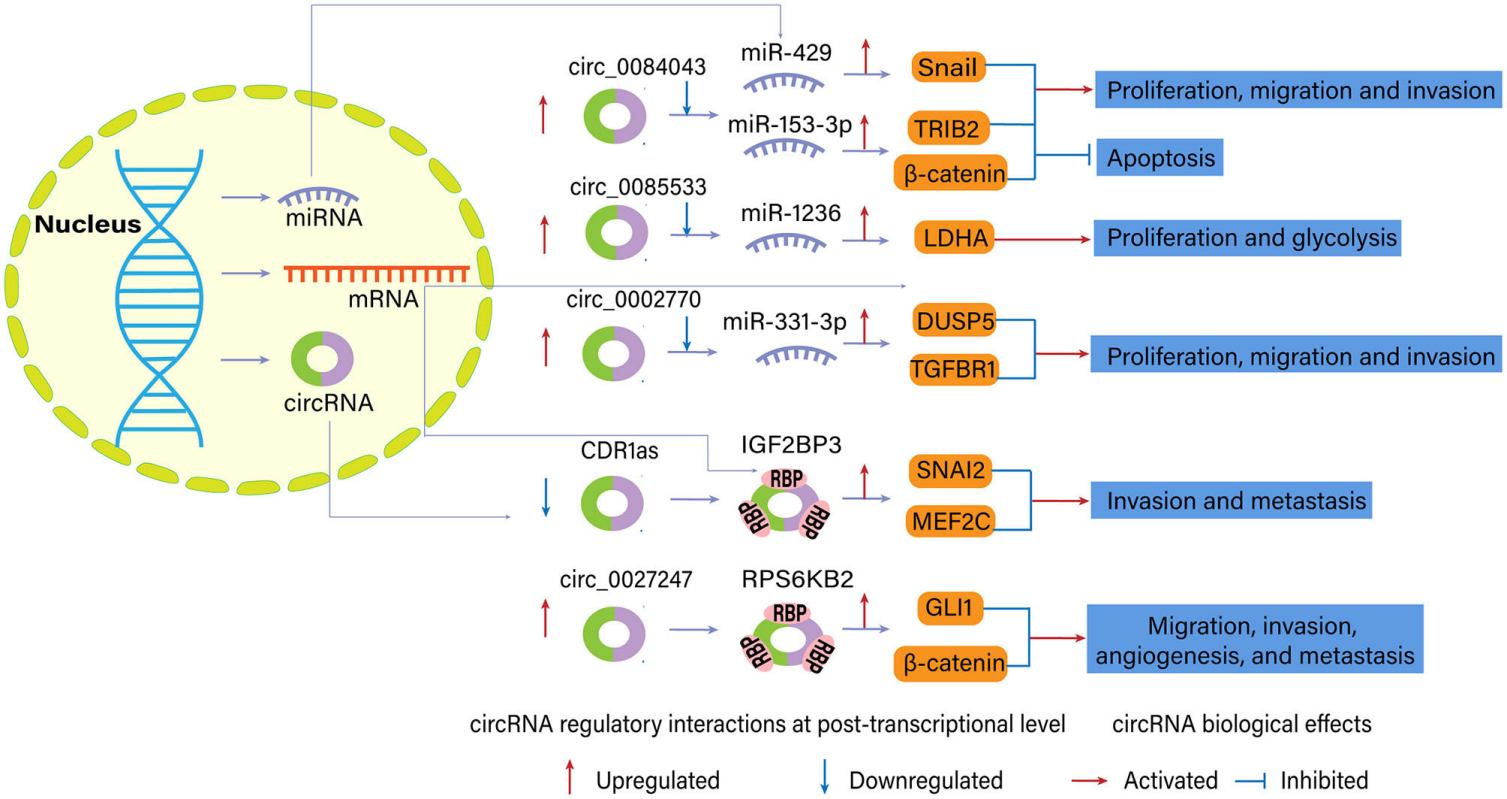
Examples of regulatory interactions and biological effects of dysregulated circular RNAs (circRNAs) in melanoma (Luan et al., 2018; Chen J. et al., 2020; Chen Z. et al., 2020; Hanniford et al., 2020; Jin et al., 2020; Qian et al., 2020).

Studies focusing on circRNAs–miRNAs sponging assessed the biological effects of circular molecules and explored regulatory networks involving the miRNAs and the subsequently targeted genes (Figure 4).

Several circRNAs played an oncogenic role in melanoma since their upregulation promoted cell progression and tumor growth. For example, circ_0083444, originating from the circularization of MTUS1 gene exons, sponged the downstream miR-622 and miR-1208 in conjunctival melanoma based on the bioinformatics algorithm. They may participate in the ErbB signaling pathway, MAPK signaling pathway, and Wnt signaling pathway to facilitate melanoma progression (Shang et al., 2019).

In another study, researchers found that the expression of circ_0084043 was elevated in melanoma tissue (Luan et al., 2018). According to the results of bioinformatics and luciferase reporter assays, circ_0084043 was the candidate to up-regulate Snail expression by interacting with miR-153-3p, promoting proliferation, invasion, and migration of melanoma cells. Co-transfection of miR-153-3p inhibitor reversed the impact of circ_0084043 knockdown on melanoma cells. Silencing of circ_0084043 notably decreased tumor growth in the melanoma xenograft model between day 12 and 21 compared with the control group. Chen Z. et al. (2020) further confirmed the oncogenic role of circ_0084043 in melanoma, which could physically and positively control tribbles homolog 2 (TRIB2) expression via sponging miR-429. Besides, knockdown of circ_0084043 inhibited Wnt/β-catenin signaling pathway via miR-429/TRIB2 axis.

Jin et al. (2020) found that upregulation of circ_0085533 (circ-MYC) promoted proliferation, glycolysis, lactate production, and lactate dehydrogenase A (LDHA) activity of MEL-CV cells via binding to miR-1236. Western blotting and luciferase activity assay reported that miR-1236 could target 3′ UTR of the LDHA gene and reduce LDHA expression. Besides, the transcriptional factor c-Myc and its target splicing factor SRSF1 could positively regulate the expression of circ-MYC, confirming the role of the c-Myc-SRSF1 axis on melanoma.

Tian et al. (2020) found that circ-FOXM1 promoted cancer progression in melanoma cells, which is consistent with findings in non-small-cell lung cancer (Liu G. et al., 2019). circ-FOXM1 manifests a sponging effect for miR-143-3p, a regulatory element of Flotillin 2 (FLOT2). FLOT2 is a cell progression and motility factor associated with lymph node metastasis and Breslow depth in melanoma. FLOT2 elevation abrogates the effects of miR-143-3p on proliferation, apoptosis, invasion, and glycolysis in melanoma cells (Tian et al., 2020).

Another circRNA with a similar mechanism in melanoma is circ_0002770, which exhibits sponging ability for miR-331-3p. circ_002770 is sharply upregulated in melanoma tissues and cell lines. Its depletion suppresses invasion, migration, and proliferation of melanoma cells *in vitro* and tumor growth *in vivo*. One study showed that mir-144 regulated MAPK signaling pathway factors, such as DUSP5 and TGFBR1 (Qian et al., 2020). Other circRNAs which also acted as oncogene and miRNA sponge in melanoma included circ_0025039 (Bian et al., 2018), circ_0016418 (Zou et al., 2019; Lu et al., 2020), circ_0020710 (circ-CD151) (Wei et al., 2020), circ_0079593 (Lu and Li, 2020), circ_0001591 (Yin et al., 2021), and circ_0119872 (Liu et al., 2021). Specific miRNA targets and downstream molecules are presented in Table 1.

There is no circRNA serving as tumor suppressor gene through the miRNA sponge mechanism based on current data. circ-ITCH might be a potential candidate, which was proved to be a tumor suppressor in melanoma and other types of cancer. Lin et al. (2019) detected the expression of circ-ITCH and GLUT1 in 56 melanomas and paired normal tissues. They reported that circ-ITCH was downregulated in melanoma tissues and affected by four clinical stages of melanoma. circ-ITCH was inversely correlated with GLUT1, which controlled the glucose transport through plasma membranes in glucose metabolism. However, they did not explore the potential miRNA in this case.

#### Sponging RBPs

circRNAs can interact with RBP to regulate gene expressions (Chen et al., 2015), as supported by the co-localization of circRNAs and RBPs (Figure 4). In this condition, circRNAs may also serve as oncogenes or tumor suppressor genes. circRNA cerebellar degeneration-related 1 antisense (CDR1as) was characterized as a candidate melanoma suppressor and a hallmark of melanoma progression, altering cell invasion *in vitro* and melanoma metastasis *in vivo* (Hanniford et al., 2020). Previous reports suggested CDR1as inhibited miR-7 function through conserved binding sites. However, CDR1as knockdown in this study had limited effects on the abundance of miR-7 and signals from a fluorescent miR-7 reporter. miR-7 depletion was insufficient to revert the increased invasion elicited by CDR1as depletion, so there should be other critical molecules functioning in the role of CDR1as in melanoma. Hanniford et al. (2020) confirmed the direct interaction between CDR1as and IGF2BP3, an oncofetal protein regulating a large series of target transcripts. IGF2BP3 and a repertoire of its targets, such as SNAI2, MEF2C, mediated invasion effects of CDR1as on melanoma cells.

Chen J. et al. (2020) identified that circ_0027247 derived from gene GLI1 (circ-GLI1) could upregulate the expression of CYR61 in melanoma cell lines via MYC-mediated manner and promote migration, invasion, angiogenesis, and metastasis of melanoma cells. Interestingly, circ-GLI1 positively affected GLI1 and β-catenin expression (terminal effector molecules in Hedgehog/GLI1 and Wnt/β-catenin pathway) at post-translational level but not translational level. RBP p70S6K2 (namely, RPS6KB2) was identified to interact with circ-GLI1 and inactivate GSK3β by phosphorylating GSK3β at Ser9. RPS6KB2 could block the binding of GSK3β to GLI1 and β-catenin proteins, inhibiting the degradation and enhancing the expression of these two proteins.

### Roles in Clinical Implications of Melanoma

Even though a small group of studies examined the relationship between circRNA expression and clinicopathological characteristics of melanoma, essential findings were obtained regarding the prognostic and predictive value of circRNAs in melanoma patients. circ_0025039 expression was significantly associated with pathological node status, pathological metastasis status, and clinical stage. Patients with higher expression of circ_0025039 in melanomas had a shorter survival time than those with lower expression (Bian et al., 2018). circ_0002770 was remarkably increased in metastatic melanoma tissue and decreased in primary tumor tissue, indicating a smaller survival time and poorer prognosis (Qian et al., 2020). circ_0001591 level in blood serum negatively correlated with overall survival and disease-free survival of melanoma patients (Yin et al., 2021).

Another example was circ_0084043, strongly associated with the tumor-node-metastasis (TNM) stage of melanoma, but not with others, for instance, age, sex, family history, and ulcer. Univariate and multivariate Cox regression analysis indicated that high expression of circ_0084043 independently predicted poor overall survival for melanoma patients (Luan et al., 2018). Depletion of CDR1as was associated with a series of prognostic factors, such as stage, ulceration, thickness, and mitotic index. Downregulation of this molecule could be related to shorter progression-free and overall survival (Hanniford et al., 2020). Melanoma cell lines with an abundance of CDR1as were less sensitive to multiple MAPK signaling pathway inhibitors but strikingly more sensitive to three GPX4 inhibitors (Hanniford et al., 2020).

Wei et al. (2020) found that the level of circ_0020710 was higher than that of the paired normal tissues and that of benign nevi tissues, with an area under the curve (AUC) of 0.692 in distinguishing melanoma tissues and normal ones. circ_0020710 expression was significantly correlated with the advanced Breslow depth and Clark level. Cox regression analysis demonstrated that the elevated circ_0020710 level, clinical stage, Breslow depth, and Clark level were independent prognostic factors in melanoma. Elevated circ_0020710 could increase the level of CXCL12 via sponging miR-370-3p. The combination of anti-PD-1 therapy and circ_0020710/CXCL12 axis inhibitor (AMD3100) significantly inhibited the growth of subcutaneous xenograft tumors compared with single-agent treatment or control treatment. These results indicated that inhibition of circ_0020710/CXCL12 axis might strengthen the therapeutic effects of anti-PD-1 therapy in melanoma patients (Wei et al., 2020).

In brief, these findings proposed that circRNAs could act as prognostic biomarkers in determining the survival time or predictive biomarkers for treatment response, which might aid the selection of treatment modalities for individual patients.

## Discussion

Recent advances in the molecular technologies of genome sequencing, transcriptome analysis and epigenetic analysis have promoted our understanding of melanomagenesis. Relevant signaling pathways, genetic alterations and epigenomic modifications are identified over the past decade (Hallajzadeh et al., 2020). MAPK pathway has the highest oncogenic and therapeutic potential with the disease. It is reported that more than 80% of benign moles have an upregulation of the MAPK pathway due to a single somatic mutation (Shain et al., 2015; Savoia et al., 2019). Genetically, the progression cascade of melanoma is started by mutations known to activate the MAPK pathway (Shain et al., 2015). Another remarkable pathway in melanoma development is the Wnt/β-catenin signaling pathway, which is found to be active in about 30% of melanoma cases (Xue et al., 2016). The canonical Wnt/β-catenin signaling pathway plays a crucial role in melanocyte development by activating microphthalmia-associated transcription factor (MITF) (Larue and Delmas, 2006). MITF, in turn, modifies melanocyte proliferation, differentiation, motility, survival and other physiological processes by regulating the transcription of various genes and multiple signaling factors, such as cAMP, PKC, MEK, and β-catenin (D'Mello et al., 2016). Other dysregulated signaling pathways, such as Ras/Raf, PI3K/Akt/mTOR, and NIK, are implicated in the loss of cellular homeostasis, promotion of epithelial-mesenchymal transition (EMT) and transition of melanocytes. One of the downstream molecules of the above-mentioned signaling pathways, nuclear factor-kB (NF-kB), is hyperactivated in melanoma and modulate the apoptosis, angiogenesis, and tumor cell invasion (Amiri and Richmond, 2005).

Diverse circRNA could facilitate melanoma development and progression through regulating or interacting with important signaling proteins in oncogenesis, ranging from components of cancer-related pathways to factors in cell cycle control and apoptosis. For example, circ_0002770 serves as an oncogene by indirectly regulating the expression of DUSP5 and TGFBR1, two of MAPK signaling pathway factors, via sponging miR-331-3p (Qian et al., 2020). circ_0027247 and cir_c0084043 crosstalk with Wnt/β-catenin pathway via interacting with miR-429 and RPS6KB2, respectively, and regulate cellular functions, such as proliferation, invasion, metastasis, and angiogenesis (Chen J. et al., 2020; Chen Z. et al., 2020). According to GO and pathway analysis, circ_0067531 is associated with activation of MAPK activity; circ_0000853 might play a potential role in NF-kappa B signaling pathway; circ_0000869 might be engaged in G-protein coupled receptor signaling pathway (Ju et al., 2018).

Cyclin-dependent kinases (CDKs) act on cell cycle progression and transcriptional regulation, and are critical regulators of DNA replication and mitosis in the cell cycle (Santo et al., 2015). CDK4/CDK6 mediates the transition from G1 to S phase by forming complexes with cyclin D. Deregulated CDK4 activity has been observed in multiple malignant tumors, therefore, it could be related to the occurrence and development of cancers. In melanoma, circ_0025039 facilitates melanoma cell growth, invasion and glucose metabolism by inhibiting CDK4 expression through sponging miR-198 (Bian et al., 2018). CXCL12, a well-studied chemokine family member, is highly produced in malignancy along with its receptor CXCR4. The CXCL12/CXCR4 axis is involved in many physiological and pathological processes, for example, embryogenesis, wound healing, angiogenesis, tumor development, and suppressive immune cell recruitment (Yin et al., 2019; Qian et al., 2020). Increased circ_0020710 is found to drive tumor progression through the miR-370-3p/CXCL12 axis. The combined therapy of AMD3100 and anti-PD-1 effectively control the melanoma growth, so the circ_0020710/ CXCL12 axis could be a potential target for melanoma treatment (Yin et al., 2019).

Several tested applications of circRNA in therapy were proposed considering the close association between circRNA and the occurrence and development of malignancy. It could be a viable approach to promote the expression of particular circRNA or inject exogeneous circRNA that sponges upregulated oncogenic miRNA or interacts with specific RBP. For example, injection of the expression vector of circ-ITCH decreases glucose intake and cell proliferation activity in melanoma cells, which offers the potentials to improve the outcomes of cancer therapy. On the other hand, many studies test in parallel miRNA mimetics/mimics or siRNA to validate the function of circRNA in gene regulation through miRNA sponging activity by competing for linear pre-mRNA splicing. As an example, circ_0084043 siRNA and miR-153-3p mimics remarkably repress the proliferative, migratory and invasive capacities of melanoma cells. circ_0084043 silencing significantly represses tumor growth in xenograft mice model (Luan et al., 2018). However, these experiments are still at an initial and exploratory stage. Additional and extensive investigations are required to verify these therapeutic options in future clinical practice.

The different expression level of circRNAs detected in tissue samples supports the essential role of circRNAs in melanoma and other diseases. The expression profile of circRNAs in human fluids, such as exosomes, blood, saliva, urine, semen, sputum and breast milk, is another auxiliary area with promising prospects that deserves further research. A recent study showed that exosomes derived from gastric cancer cells deliver ciRS-133 into preadipocytes, facilitating the transformation of preadipocytes into brown-like cells via activating PRDM16 and suppressing miR-133. Additionally, ciRS-133 knockdown reduces cachexia in tumor-implanted mice (Zhang et al., 2019). Thus, exosome-delivered circRNAs are involved in white adipose tissue and cancer-associated cachexia. In patients with oral squamous cell carcinoma, elevated circ_0000199 in circulating exosomes is positively associated with tumor size, lymphatic metastasis, TNM stage, higher recurrence and mortality rate, and poor survival outcome. Bioinformatics analysis predicts that circ_0000199 could affect multiple tumor-related signaling pathways by simultaneously interacting with miR-145-5p and miR-29b-3p (Luo et al., 2020). circRNAs appear to be highly conserved across mammals, and exosomal circRNAs are detected in bovine milk by high-throughput RNA sequencing analysis. The conserved nature might confirm the role of circRNAs in biological functions (Wang Y. H. et al., 2018). Exosomal circRNAs can regulate signaling pathways and participate in cell proliferation, tumor metastasis, and drug resistance progression in cancer (Wang et al., 2019). Since exosomes have an important role in intercellular communication, unraveling the roles of exosomal circRNAs may be helpful in clinical diagnostics and therapeutic development.

Salivary is the most accessible and non-invasive human body fluid, probably harbor biomarkers for many diseases and cancers. Bahn et al. (2015) identified over 400 circRNAs in human cell-free saliva from healthy individuals using high-throughput RNA sequencing. Results of this study showed that salivary circRNAs may be implicated in intercellular signaling and inflammatory response. Since cancer progression could be related to inflammation, salivary circRNAs may also participate in tumorigenesis. circRNAs are actively involved in tumor-related biological events and emerge as promising biomarkers for personalized medicine and non-invasive detection of diseases. However, circRNAs in exosomes, salivary, blood, and other accessible body fluids have not been extensively studied in melanoma patients. Supplementary researches may be required to investigate the expression level and molecular mechanisms of circRNAs in body fluid from cancer patients, which paves the way for functional, biological, and biomarker discoveries.

There is tremendous potential in the function of circRNAs in the field of cancer intervention, diagnosis, and treatment. However, for better mechanism investigations and clinical practice, the following limitations and challenges in circRNA research need to be addressed. Firstly, current studies directly conclude that circRNAs function as sponges or encoding genes to regulate cancer development, while the data on prerequisites of particular mechanisms are not provided. And identified circRNAs might not have important functions or functions, not through the currently proposed mechanism (Li et al., 2020). For example, some circRNAs that participate in tumorigenesis might not function through the proposed mechanisms as miRNA sponge. Of note, miRNAs have a much higher amount than circRNAs, thus, the ratio of circRNA to miRNA should be considered in further research to exclude any collaborative, competitive, or decoy regulatory process. The ratio should be sufficiently high for miRNA sponge as a reliable mechanism. Secondly, the current terminology of circRNAs is not systematic which causes problems for both experimental and bioinformatics research. Maybe a standard nomenclature of circRNA, in which the host gene, the chromosome location, and the arbitrary number of circRNAs are combined, will facilitate the communications among researchers (Liu M. et al., 2019). A well-constructed circRNA database with a naming system and comprehensive information will be helpful. Thirdly, all dysregulated circRNAs in cancer patients may be used as biomarkers for diagnosis and prognosis, however, the sensitivity and specificity of circRNAs are not currently assessed in many studies, bringing difficulties to real-world applications. Maybe ROC analysis can be performed to select the most effective biomarkers for disease. Moreover, circRNAs can be involved in many tissues and diseases, thus, targeting circRNAs as a treatment strategy for cancerous cells may cause inadvertently pathological damage to normal cells and tissues. Future studies should take the risk of off-target effects on the whole body and organs into account and improve the specificity of circRNA target treatment. Last but not least, the copy number of some circRNAs may be low in target cells, and the abundance of circRNAs should be enriched to improve the efficacy of circRNA-based cancer therapy. It matters that to find the most efficient delivery system of circRNAs based on different types of target cells or synthesize circRNAs with more miRNA binding sites and higher efficacy.

Although the relativity of circRNAs to cancer has attracted increased attention during recent years, a systematic review declaring the potential roles of circRNAs in melanoma pathogenesis and clinical implications is yet to be published. There is a significant difference in the expression level of circRNAs in melanoma samples and healthy controls. This study reviews several mechanisms for circRNAs function in melanoma. circRNAs function in melanoma by miRNA sponging and RBP binding, ultimately controlling several important signaling pathways and cancer-related cellular processes, including proliferation, migration, invasion, metastasis, apoptosis, and glucose metabolism. The expression level of circRNAs could be correlated with the TNM stage, Breslow depth, survival time and other prognostic factors. circRNAs may predict the response of melanoma patients to specific drugs and guide the selection of therapeutic options for individual patients, such as MAPK signaling pathway inhibitors and GPX4 inhibitors. Several limitations should be focused on in this study. A major problem of the reviewed articles is technical issues, which result from the deficient expression of circRNAs and the lack of standardized protocol for evaluation of these transcripts. The other limitation is the relatively small number of studies and samples. Besides, most of the described mechanisms in order to actually provide any clinical utility need to be validated in future studies.

Compared with mRNAs, long ncRNAs, and miRNAs, studies of circRNAs in melanoma are just at the beginning. The underlying mechanism of circRNAs and their exact functions in melanoma has not been completely understood. For the forthcoming studies, it is urgent to show how circRNAs orchestrate the melanoma regulatory networks and how this may prove useful in future treatment strategies. Besides, the roles of circRNAs in exosomes and human body fluid from melanoma patients remain unclear. Some scholars report that circRNAs may be closely connected to tumor stage, cell developing environments and different tumor subtypes (Jahani et al., 2020), and these results could be further verified in melanoma tissues. circ_0016418 is revealed to influence the EMT signaling pathway of skin melanoma cells by measuring the expression of E-cadherin, N-cadherin, and Vimentin (Zou et al., 2019). EMT occurs in embryonic development and is considered the symbol of carcinogenesis and metastasis. Investigations on cicRNAs regulation in EMT may open up new perspectives for researchers to decipher the mechanism of melanoma development. For therapeutic applications, more studies should focus on the sensitivity and specificity of circRNAs applied in the melanoma population, and compare their accuracy as biomarkers for melanoma. More efforts are warranted to design an accurate and effective delivery system of si-circRNAs or circRNAs with minimal side effects, or to develop artificial circRNAs specific for each pathological condition with more sponging sites for miRNA than natural ones.

## Conclusion

This systematic review summarizes the biogenesis, functions, expression patterns of circRNAs in melanoma. These circRNAs work as miRNA sponges or interact with RBPs to regulate the expression level of target miRNA or proteins in melanoma, which finally control the multiple cellular signaling pathways and cancer-associated cellular transitions. Given the characteristics of high stability, specificity, and involvement in gene regulation, circRNAs are expected to be potential novel biomarkers for diagnosis, prognosis, and treatment of melanoma.

## Data Availability Statement

The original contributions presented in the study are included in the article/Supplementary Material, further inquiries can be directed to the corresponding author.

## Author Contributions

KT and HZ reviewed articles, collected data, and wrote the main manuscript text. KT and YL prepared figures and tables. QS critically analyzed the data. HJ designed the work and critically revised it for important intellectual content. All authors reviewed the manuscript and approved it for publication.

## Conflict of Interest

The authors declare that the research was conducted in the absence of any commercial or financial relationships that could be construed as a potential conflict of interest.
