# Supplementary material for: Circular RNA as a Potential Biomarker for Melanoma: A Systematic Review
Source: Front Cell Dev Biol. 2021 Apr 1;9:638548. doi: 10.3389/fcell.2021.638548 (PMC8047128; doi:10.3389/fcell.2021.638548)
Supplement: Supplementary file 1 [file Table_1.DOCX]

**Supplemental Table 1. Quality assessment of the included studies.**

| Study | Selection |  |  |  | Comparability | Exposure |  |  | Scores |
| --- | --- | --- | --- | --- | --- | --- | --- | --- | --- |
|  | Adequate definition of cases | Representative-ness of the cases | Selection of  controls | Definition of  controls | Control for important factor | Ascertainment of exposure | Same method  to ascertain for cases and controls | Non-Response  rate |  |
| Yang et al.,  2018 | ★ | ★ | _ | ★ | ★ | ★ | ★ | ★ | 7 |
| Ju et al.,  2018 | ★ | ★ | ★ | ★ | ★ | ★ | ★ | ★ | 8 |
| Luan et al.,  2018 | ★ | ★ | ★ | ★ | ★ | ★ | ★ | ★ | 8 |
| Bian et al., 2018 | ★ | ★ | ★ | ★ | ★ | ★ | ★ | ★ | 8 |
| Shang et al., 2019 | ★ | _ | ★ | _ | ★ | ★ | ★ | ★ | 6 |
| Lin et al., 2019 | ★ | ★ | ★ | ★ | ★ | ★ | ★ | ★ | 8 |
| Zou et al., 2019 | ★ | ★ | ★ | ★ | ★ | ★ | ★ | ★ | 8 |
| Wei et al., 2020 | ★ | ★ | ★ | ★ | ★★ | ★ | ★ | ★ | 9 |
| Qian et al.,  2020 | ★ | ★ | - | - | ★ | ★ | ★ | ★ | 6 |
| Tian et al.,  2020 | ★ | ★ | ★ | ★ | ★ | ★ | ★ | ★ | 8 |
| Chen et al.,  2020 | ★ | ★ | ★ | ★ | ★ | ★ | ★ | ★ | 8 |
| Jin et al.,  2020 | ★ | ★ | ★ | ★ | ★ | ★ | ★ | ★ | 8 |
| Hanniford et al., 2020 | ★ | ★ | ★ | ★ | ★ | ★ | ★ | ★ | 8 |
| Lu R et al., 2020 | ★ | ★ | ★ | ★ | ★ | ★ | ★ | ★ | 8 |
| Yin et al., 2020 | ★ | ★ | - | ★ | ★ | ★ | ★ | ★ | 7 |
| Lu J et al.,  2020 | ★ | ★ | - | ★ | ★ | ★ | ★ | ★ | 7 |
| Liu et al.,  2021 | ★ | _ | ★ | _ | ★ | ★ | ★ | ★ | 7 |
